# The single nucleotide β -arrestin2 variant, A248T, resembles dynamical properties of activated arrestin

**DOI:** 10.3906/kim-1910-46

**Published:** 2020-04-01

**Authors:** Özge ŞENSOY

**Affiliations:** 1 Department of Computer Engineering, The School of Engineering and Natural Sciences, İstanbul Medipol University, İstanbul Turkey

**Keywords:** Arrestin, G protein-coupled receptor, phosphorylation-independent activation, cancer, single nucleotide polymorphism, molecular dynamics simulation

## Abstract

β -arrestins are responsible for termination of G protein-coupled receptor (GPCR)-mediated signaling. Association of single nucleotide variants with onset of crucial diseases has made this protein family hot targets in the field of GPCR-mediated pharmacology. However, impact of these mutations on function of these variants has remained elusive. In this study, structural and dynamical properties of one of β -arrestin2 (arrestin 3) variants, A248T, which has been identified in some cancer tissue samples, were investigated via molecular dynamics simulations. The results showed that the variant underwent structural rearrangements which are seen in crystal structures of active arrestin. Specifically, the “short helix” unravels and the “gate loop” swings forward as seen in crystal structures of receptor-bound and GPCR phosphopeptide-bound arrestin. Moreover, the “finger loop” samples upward position in the variant. Importantly, these regions harbor crucial residues that are involved in receptor binding interfaces. Cumulatively, these local structural rearrangements help the variant adopt active-like domain angle without perturbing the “polar core”. Considering that phosphorylation of the receptor is required for activation of arrestin, A248T might serve as a model system to understand phosphorylation-independent activation mechanism, thus enabling modulation of function of arrestin variants which are activated independent of receptor phosphorylation as seen in cancer.

## 1. Introduction

G protein-coupled receptors (GPCRs) are responsible for communication of the cell with surrounding environment. Upon ligand binding the receptor undergoes a set of conformational changes which is recognized by heterotrimeric G protein. Subsequently, G protein is activated, and the alpha subunit of the protein is dissociated from the beta-gamma dimer, each of which can initiate discrete signaling pathways. Depending on the type of the ligand certain cytosolic residues of the GPCR is phosphorylated by corresponding GPCR kinases. Subsequently, a protein which is responsible for GPCR-mediated signal termination, namely arrestin, is recruited to the membrane and then it binds to the receptor. Consequently, occupation of the cytosolic region of the receptor by arrestin, which is otherwise occupied by the G protein, leads to desensitization [1]. Finally, receptor-arrestin complex is internalized and depending on the needs of the cell it is either recycled back to the membrane or directed to lysosome for degradation [2,3].

**Figure 1:**
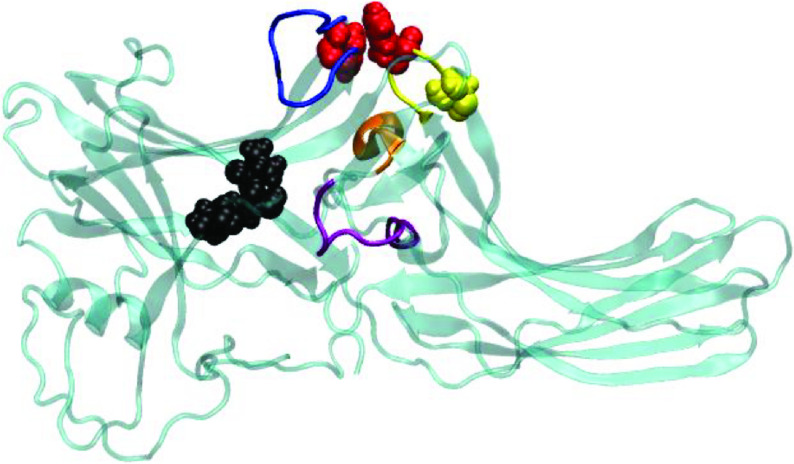
Structural depiction of the inactive β -arrestin2 (PDB ID:3P2D, chain B (Zhan et al., 2011). The regions involved in the activation mechanism are color-coded. The “C loop”, the “short helix”, the “gate loop”, the “aromatic core”, and the “finger loop” are shown with yellow, orange, purple, red and blue. The residues that make up the “polar core” are shown in black and van der Waals representation. The 248th residue is shown in yellow and van der Waals presentation.

Arrestin protein family consists of four members, namely arrestin 1, 2(β -arrestin1), 3(β -arrestin2), and 4. In spite of sharing a high degree of sequence similarity and conserved structural fold, which is composed of two β -sheet sandwich domains (N- and C-) that is connected by a linker (Figure 1), the members display remarkable differences in their preferences for phosphorylation of the receptor, which is needed for formation of high affinity arrestin-receptor complex. Arrestin 1 and 4 are known as visual arrestins and can only bind to activated and phosphorylated rhodopsin, whereas β -arrestin1 (arrestin 2) and β -arrestin2 (arrestin 3) can interact with various GPCRs. Interestingly, β -arrestin2 can also bind to nonphosphorylated GPCRs depending on the type of the receptor [4]. Here, the formation of the high-affinity GPCR-arrestin complex is shown to proceed through a “two-step” mechanism [5-7]. According to that, first, N-domain of arrestin interacts with the receptor-attached phosphates which leads to removal of structural constraints that stabilize the inactive conformation of arrestin. In the second step, the high-affinity complex is formed between arrestin and activated/phosphorylated receptor by engagement of key structural elements that are exposed in the first step. Here, it is important to emphasize that the most remarkable conformational change that occurs in arrestin upon activation is the relative rotation of the C-domain with respect to the N-domain by about 17–20°[8]. Among the structural constraints that stabilize the inactive conformation of arrestin, the “polar core”, which is located on the N-domain, is wellestablished. It is composed of five closely packed charged residues and it participates in the stabilization of the C-terminal tail of arrestin. It is released upon binding to activated/phosphorylated receptor as a result of disruption of the charge balance in the “polar core” [5]. Since the “polar core” is involved in activation mechanism of arrestin, destabilization of the region leads to formation of constitutively active phenotypes which do not require receptor phosphorylation for formation of high-affinity arrestin-receptor complex [9]. Moreover, the “gate loop” (residues 295-306) [10,11], the “C-loop”(residues 244-249), the “short-helix” (residues 313-317), and the “aromatic core” have been shown to undergo conformational rearrangement upon activation as evident from comparison of crystal structures of inactive and active arrestin [12,13]. These regions have been also proposed to be involved in the activation mechanism [14] (Figure 1), while the “finger loop” (residues 66-75) is primarily required for binding to the receptor [11,15]. As such, any mutation occurring in these regions might prevent either activation of arrestin or receptor binding. Alternatively, it might also help removal of the structural restraints that stabilize the inactive conformation of arrestin, thus triggering activation of the protein in the absence of phosphorylated receptor. The single nucleotide β -arrestin2 variant, namely A248T mutant (COSMIC-MUTATION-ID: COSM4749050) is identified in some intestine and stomach cancer tissue samples and shown to have a high pathogenic score (0.99) according to the FATHMM prediction [16]. Therefore, presumably, it can be given as an example to the latter, where arrestin is thought to be activated without interacting with phosphorylated receptor. In this study, structural and dynamical properties of A248T were explored by means of atomistic molecular dynamics simulations (total of 6 μs). The results were discussed considering available experimental data and compared to those which are obtained from molecular dynamics simulations of wild-type arrestin 1 and wild-type β -arrestin2 that were used as the inactive and activated representatives of arrestin, respectively.

## 2. Materials and methods

For wild type arrestin1, the D chain of the crystal structure with PDBID: 1CF1 [17] was used whereas for wild-type arrestin3 the B chain of the crystal structure with PDBID: 3P2D [18] was used. The missing regions of the crystal structures were modeled using Swiss-Modeler [19]. The variant was modeled using Pymol [20]. The protonation states of residues were determined using the PROPKA methodology at pH 7.0 [21,22]. All MD simulations were performed with Gromacs Package (version 5.1.4) [23] and CHARMM36 force field [24] was used to model the protein. TIP3P [25] was used to model water molecules. Temperature was kept constant at 310 K using Berendsen thermostat with coupling time of τ T= 0.1 ps. For constant pressure simulations, Berendsen barostat [26] was used with a coupling time of τ P= 0.5 ps. The particle mesh Ewald method (PME) [27] was used for electrostatic interactions with a real space cutoff of 1.0 nm and a grid spacing of 0.12 nm. Lennard-Jones interactions were calculated with a twin-range cutoff scheme of 1.0 and 1.4 nm. A time step of 2 fs was used for the integration. Prior to production MD runs, systems were energy minimized via steepest descent algorithm. After the equilibration phase, the systems were simulated twice, each of which was simulated for 1 μs starting with a different initial velocity distribution. The analysis results were presented as a combination of these two replicas. MD simulations were performed using the SHAKE algorithm [28]. Outputs were saved every 20 ps. All the systems were neutralized in 0.15 M NaCl. All the analyses of the MD trajectories were carried out after removing the first 50 ns, when a relatively stable plateau was reached in backbone root-mean-square-deviations (RMSD). The rotation analysis was made as explained in detail in [14]. The secondary structures were assigned using the STRIDE algorithm [29] implemented in VMD [30]. RMSDs of the “C-loop”, the “short-helix”, the “gate loop”, and the “finger loop” were calculated by including residues 243-248, 313-317, 296-316, 65-75 (248-253, 318-322, 301-321, 70-80 in arrestin1), respectively. The integrity of the “aromatic core” was investigated by calculating the distance between the Cα atoms of F76 and F245 in β -arrestin2 (F79 and Y250 in arrestin1). The status of the “polar core” was evaluated by measuring the distance between the Cα atoms of R170 and F391 in β -arrestin2 (R175 and in F380 arrestin1). The essential dynamics analysis of the MD trajectories was carried out using g_covar and g_anaeig modules [31] of Gromacs package [23] by aligning the trajectory on the Cα atoms of the starting conformation. The covariance matrix was calculated by including only Cα atoms.

## 3. Results

In this study, atomistic molecular dynamics simulations were performed on single nucleotide variant of β - arrestin2, A248T mutant as described in Section 2. The results were discussed in light of corresponding crystal structures of arrestin. Moreover, molecular dynamics simulations were also performed on wild-type arrestin1 and β -arrestin2 to examine the impact of the mutation on dynamics of the variant and determine its activation state.

### 3.1. Exchange of alanine with bulkier threonine on the “C-loop” increases the flexibility of the “short helix” and the “gate loop”

**Figure 2:**
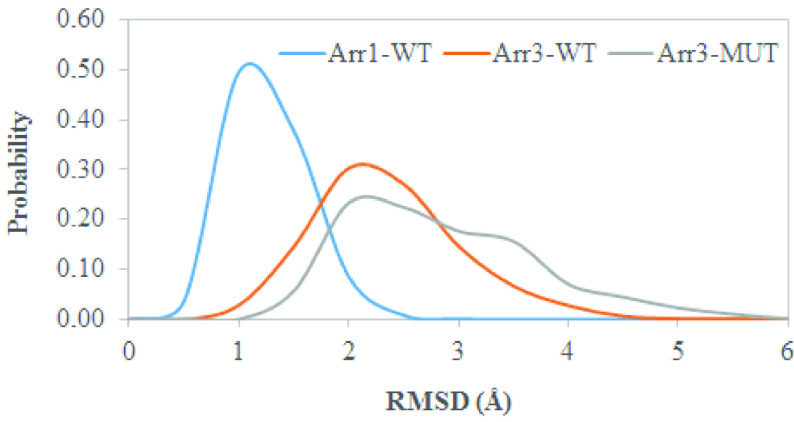
Probability distribution of RMSD of the “Cloop” obtained from molecular dynamics trajectories of the systems studied.

**Figure 3:**
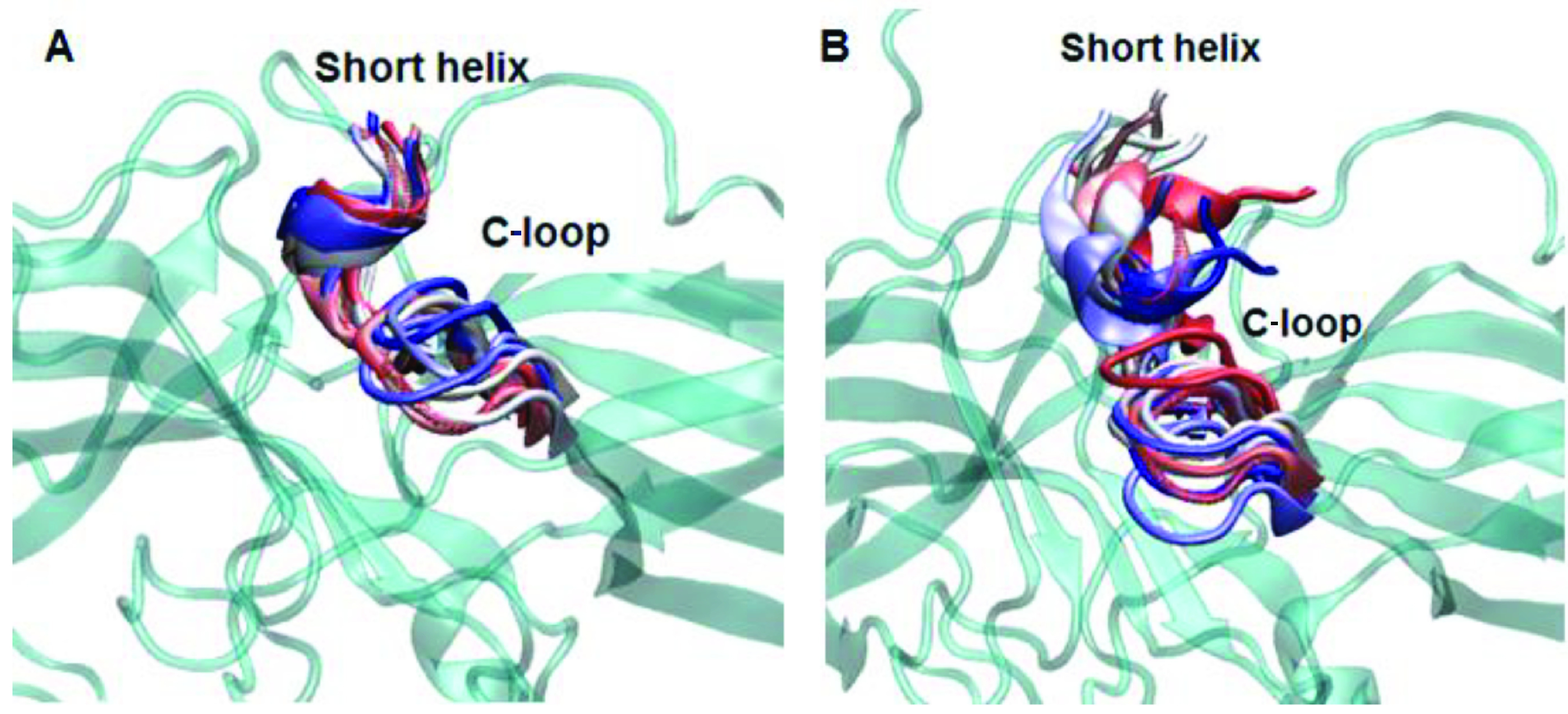
Dynamics representation of the C-loop throughout the simulation in A. WT and B. mutant protein. 3D structure of the protein is shown in cyan and cartoon representation. The short helix and the C-loop are colored with respect to the frame number: from red to blue–the number of the frame increases.

**Figure 4:**
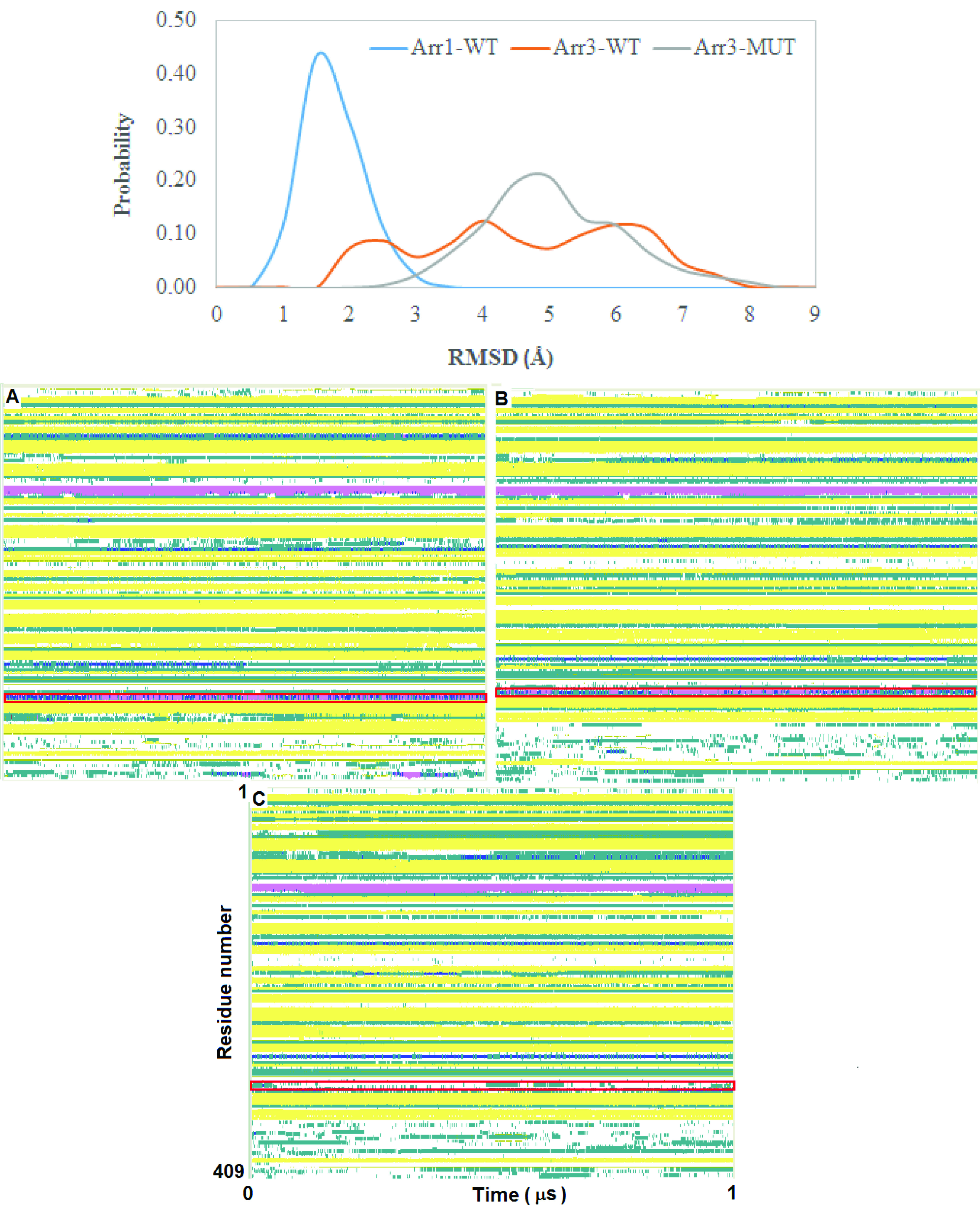
Probability distribution of RMSD of the “short helix” obtained from molecular dynamics trajectories of the systems (top). Time-line plots of secondary structures of A. wild-type arrestin1, B. wild-type β -arrestin2, C. A248T mutant. α-helix, 3-10 helix, turn, beta-sheet, and coil are shown with pink, blue, green, yellow, and white color, respectively. The residues that are located on the “short helix” are indicated with red rectangle.

**Figure 5:**
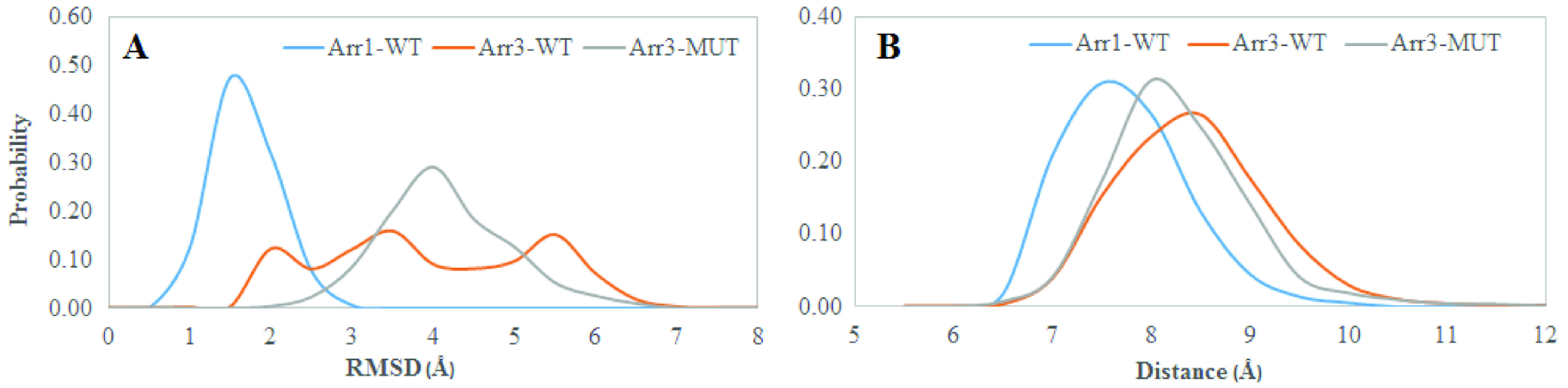
Probability distributions of A. RMSD of the “gate loop”, B. distance which is measured between the Cα atoms of F76 and F245 in β -arrestin2, (F79 and Y250 in arrestin1) which make up the “aromatic core”, obtained from molecular dynamics trajectories of the systems.

**Figure 6:**
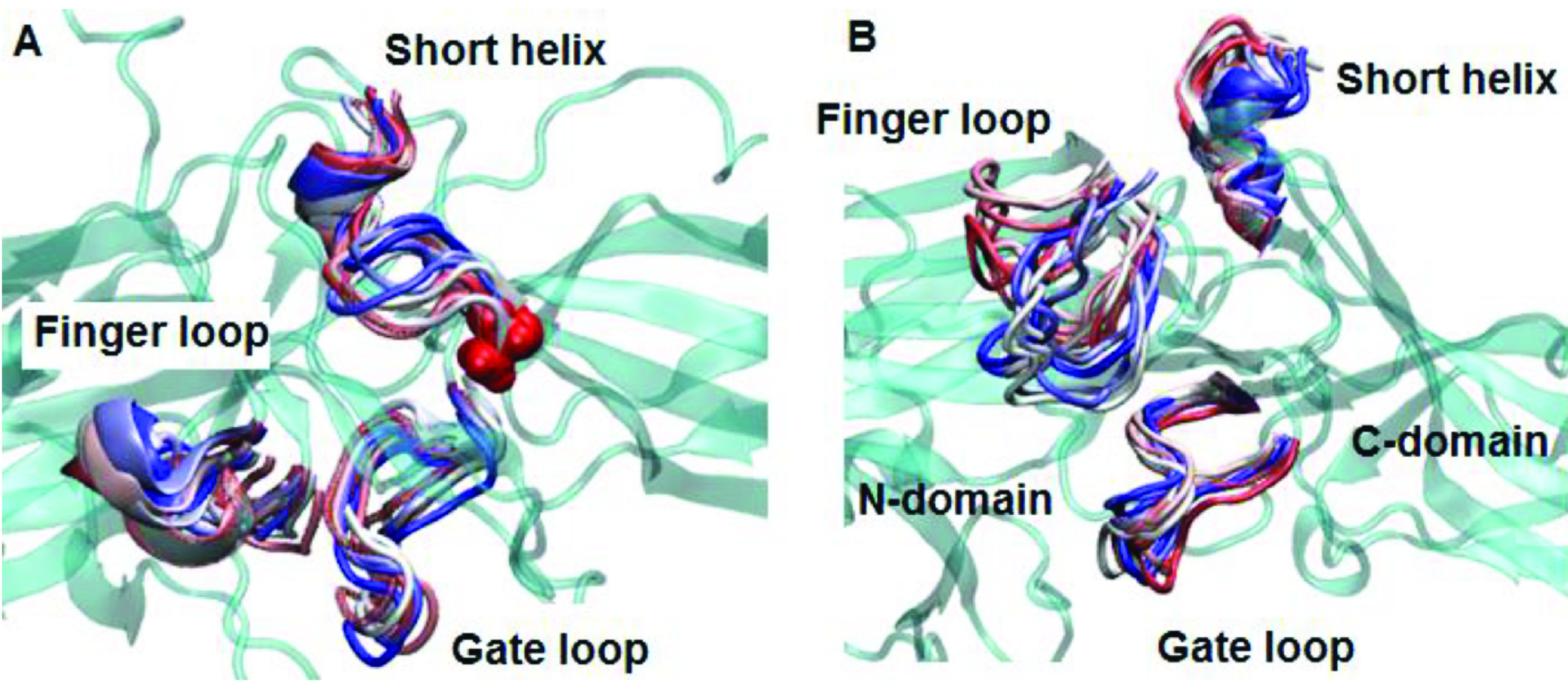
Dynamics representation of “finger loop”, “short helix” and “gate loop” throughout the simulation in A. WT and B. mutant protein. 3D structure of the protein is shown in cyan and cartoon representation. The three regions are colored with respect to the frame number: from red to blue – the number of the frame increases.

The 248th residue is located on the “C-loop” and residues on this region are in contact with the “short helix”, which is located underneath the “C-loop” as shown in Figure 1. The exchange of alanine with bulkier threonine increases the flexibility of the “C-loop” as revealed by higher RMSD values in the variant as shown in Figure 2. Consequently, this causes “C-loop” to move away from the “short helix” as shown in Figure 3. This dynamical change is in agreement with an experimental study which showed that dynamics of the “C-loop” changed upon receptor binding [32]. Interestingly, wild-type β -arrestin2 also displayed higher flexibility in the absence of any perturbation. As such, this led to detachment of the loop from the protein in the variant as also seen in crystal structures of receptor-bound and GPCR-phosphopeptide-bound arrestin (PDB IDs: 5W0P and 4JQI, respectively) [12,13]. Moreover, higher flexibility, which was observed for the “C-loop”, was also transmitted to the “short helix” as revealed by higher RMSD values pertaining to that region as shown in Figure 4. Consequently, this caused “short helix” to unravel in the variant, which is evident from loss of α-helix secondary structure. Also, this observation is in line with what is observed in crystal structures of active arrestin [12,13]. On the other hand, the “short helix” did not completely unravel in wild-type arrestin1 and β -arrestin2 as revealed by dominance of pink and blue colors which represent α-helix and 3−10-helix, respectively as shown in Figure 4. Importantly, the conformational state of the “short helix” might impact the domain rotation angle in these constructs [14] and it is discussed in detail below. We also explored the stability of the “gate loop”, which is connected to the “short helix”. The results showed that the perturbation introduced by the mutation on the “C-loop” was transmitted to the “gate loop” as revealed by higher RMSD values for the variant and wild-type β−arrestin2 but not for wild-type arrestin 1 (Figure 5A). On the other hand, the mutation did not perturb the integrity of the “aromatic core” in the variant but caused sampling of slightly longer distances which is quantified by the distance measured between Cα atoms of F76 and F245 that make up the core as shown in Figure 5B. However, the same distance which was measured between corresponding residues of arrestin1 was smaller than that of inactive β−arrestin2 (PDB ID: 3P2D) [18] which was measured as 7.2 Å. The change in dynamics of this region is consistent with experimental data [11, 33] and also with crystal structures of receptor-bound and GPCR phosphopeptide-bound arrestin (PDB IDs: 5W0P and 4JQI, respectively) [12, 13] where the “gate loop” swings forward and comes closer to the N-domain as shown in Figure 6.

### 3.2. The “finger loop” samples “upward position” in the single nucleotide variant of β -arrestin2

**Figure 7:**
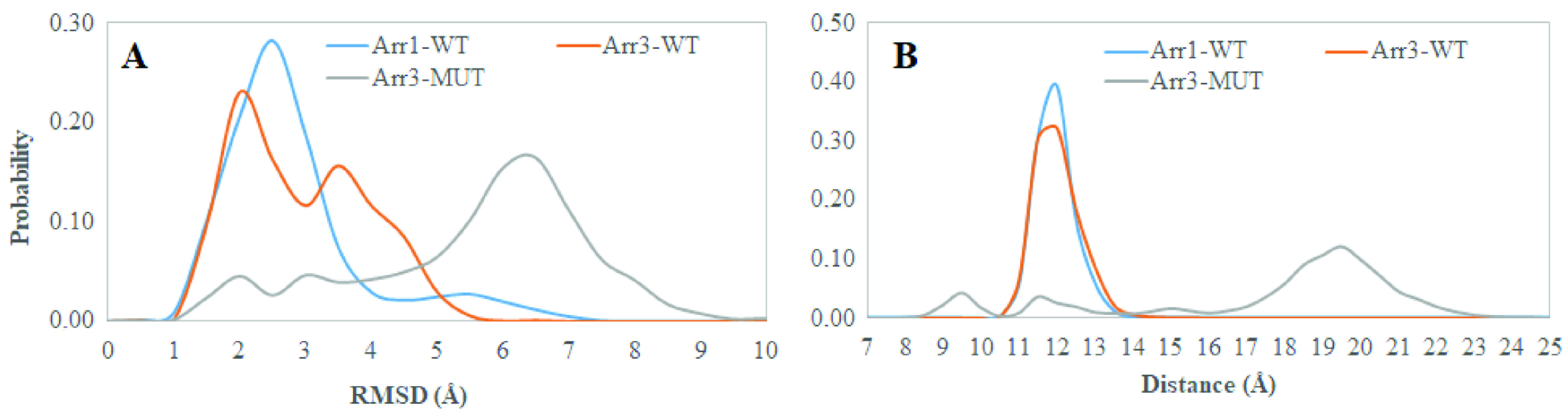
Probability distributions of A. RMSD of the “finger loop”, B. distance which is measured between Cα atoms of C60 and D70 in β -arrestin2 (A64 and M75 in arrestin1) obtained from molecular dynamics trajectories of the systems.

**Figure 8:**
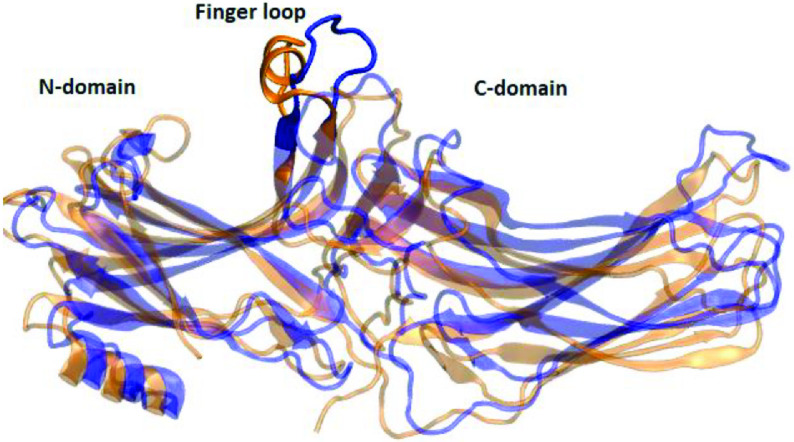
Structural alignment made between the crystal structure of receptor-bound arrestin (PDB ID: 5W0P) and a representative structure picked up from the trajectory. The proteins are shown in new cartoon representation. The crystal structure is shown in orange whereas the representative structure is shown in blue.

As mentioned above the “finger loop” has been shown to be required for binding to receptor. Moreover, the crystal structures have revealed that the loop forms close contact with the N-domain in the inactive form (PDB ID: 3P2D) [18] whereas this contact is disrupted upon activation [34] which leads to displacement of the loop from the N-domain as revealed by crystal structure of constitutively active arrestin1 mutant – R175E, receptor-bound arrestin1 and GPCR-phosphopeptide-bound arrestin (PDB IDs: 4ZRG, 5W0P, 4JQI, respectively) [10,12,13]. However, the “finger loop” adopts α-helical conformation only when arrestin is bound to the receptor as revealed by the crystal structure of the receptor-arrestin complex (PDB ID: 5DGY, 5W0P) [13, 35]. The aromatic residues (Y64 and F76) that are located at the base of the “finger loop” form contact with F245 in β -arrestin2 which is located on the “C-loop”. In our trajectories, higher flexibility was observed for the “finger loop” in the variant (Figure 7A), which consequently caused the loop to displace from the N-domain as revealed by longer distance values which were measured between the Cα atoms of C60 and D70 in β -arrestin2 (A64 and M75 in arrestin1) whereas the loop was in close contact with the N-domain both in wild-type arrestin1 and β -arrestin2 as shown in Figure 7B. Here, the loop did not adopt any secondary structure as opposed to seen in the crystal structure of receptor-bound arrestin complex (PDB IDs: 5DGY, 5W0P) [35,13] (Figure 8).

### 3.3. The single nucleotide variant can adopt active-like rotation angle without perturbing the “polar core”

**Figure 9:**
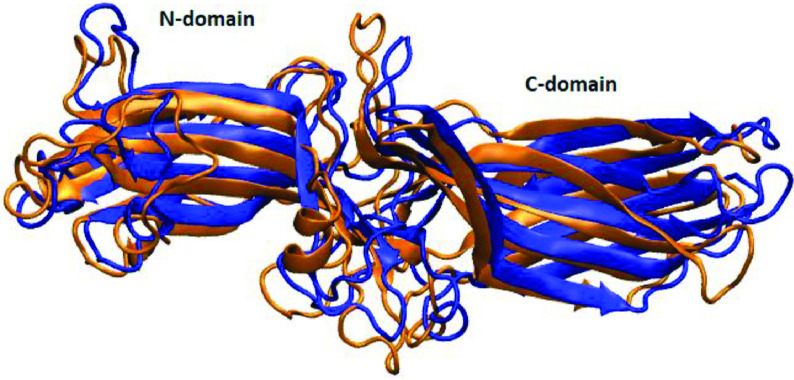
Structural alignment made between the crystal structure of receptor-bound arrestin (PDB ID: 5W0P) and a representative structure picked up from the trajectory. The proteins are shown in new cartoon representation. The crystal structure is shown in orange whereas the representative structure is shown in blue.

**Figure 10:**
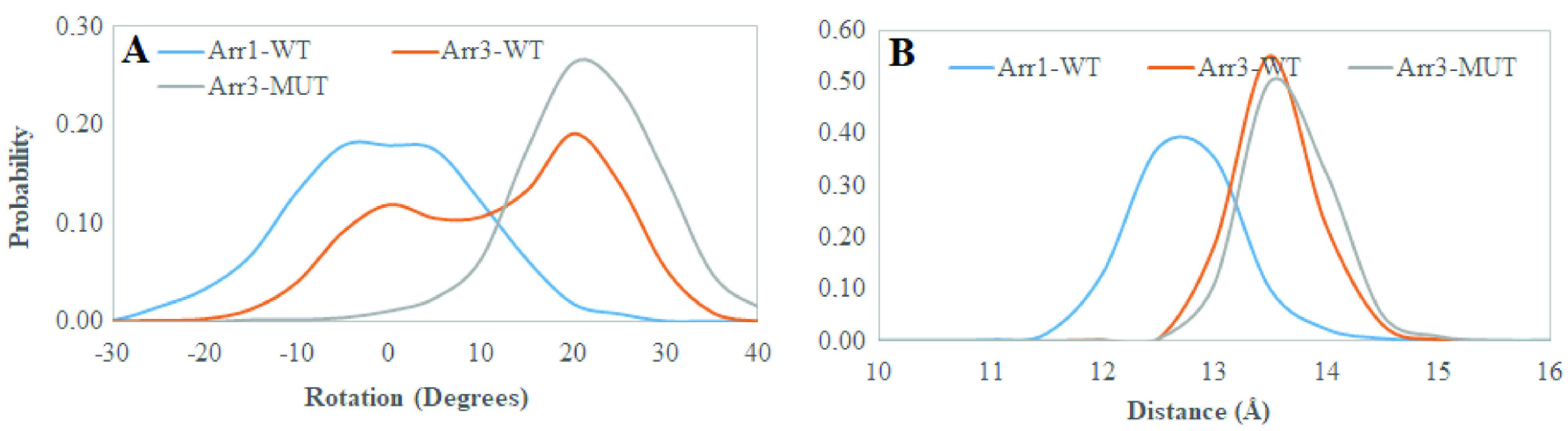
Probability distributions of A. domain rotation angle, B. distance which is measured between Cα atoms of R170 and F391 in β -arrestin2 (R175 and in F380 arrestin1) obtained from molecular dynamics trajectories of the systems.

As mentioned above the interaction of arrestin with phosphorylated C-tail of the receptor triggers the release of the structural constraints that stabilize the inactive conformation of arrestin. The “polar core” acts as the first contact point between arrestin and the phosphorylated receptor and the disruption of the charge balance within that region causes release of the C-tail of arrestin which further triggers domain rotation. After investigation of the abovementioned local structural properties, their impact on the global dynamic properties and hence the activation states of the systems were also examined. To do so, the domain rotation angle was calculated, and results showed that the variant could adopt active-like rotation angle, which is around 20°, as revealed by structural alignment made using the crystal structure of receptor-bound arrestin (PDB ID: 5W0P) and a representative structure picked up from the trajectory (Figure 9). Here, it is also important to emphasize that the domain rotation in mutant Arr3 is achieved without perturbation of the “polar core” as revealed by the intact C-terminal of arrestin as shown in Figure 10. Interestingly, wild-type β -arrestin2 could sample both inactive and active-like domain rotation angles. The results suggested that the variant could be activated independent of phosphorylation of the receptor; hence, it might resemble “activated” arrestin. On the other hand, arrestin1 sampled the domain rotation angle which resembled that of crystal structure of inactive arrestin (PDBID: 3P2D) [18], which is on average 0°, in the absence of any perturbation. This is in correspondence with the phosphorylation requirement of this subtype to get activated.

### 3.4. The “short helix” and the “finger loop” emerge in the dominant collective motions in the single nucleotide variant

**Figure 11:**
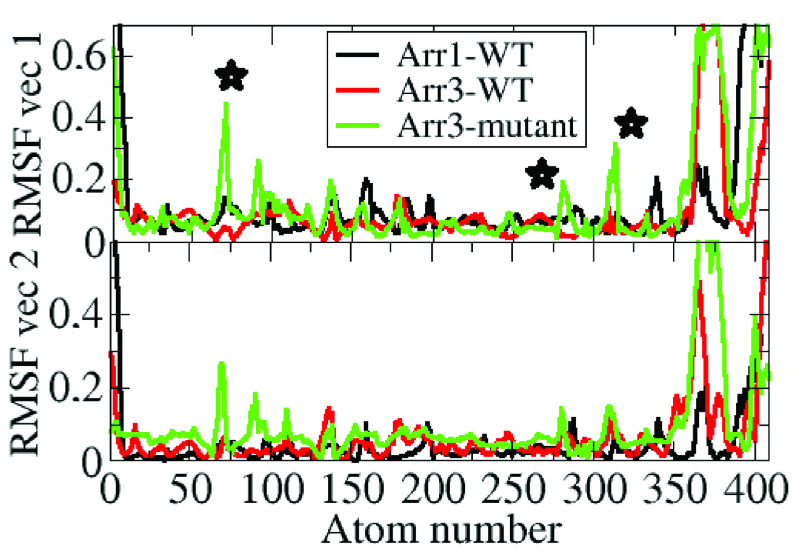
Profile of fluctuations along the first two essential eigenvectors of the systems. Important regions (“finger loop”, “gate loop”, and “short helix”) are indicated with star on the plot.

In order to investigate the dominant collective motions in the systems studied essential dynamics analysis was made. The first two eigenvectors were considered as they represented together 50% of the overall motion. The “short helix” and the “finger loop” were shown to emerge in the dominant collective motions in the variant as shown in Figure 11.

As shown above, higher RMSD values were observed both for the “short helix” and the “finger loop” in the β -arrestin2 variant. Moreover, the “finger loop” was also shown to be found in the upward position. Because unraveling of the “short helix” triggers domain rotation and the upward position of the “finger loop” is required for binding to the receptor the variant seems to resemble the “activated” state, which is in accordance with the results of the rotation angle analysis.

## 4. Discussion

The largest and the most diverse group of membrane receptors, namely GPCRs, maintain communication among the cells. Therefore, they are involved in almost all types of physiological processes in the organism. As such, any problem occurring in this protein family ends up with the onset of various crucial diseases. Recently, the discovery that arrestins also initiate G protein-independent signaling pathways has made this family one of the hot targets in the GPCR field. Moreover, association of single nucleotide arrestin variants with variability in drug response has further increased the impact of this small protein family in the field of GPCR-mediated pharmacology. However, the lack of mechanistic insight on how these single nucleotide variations impact the function of the target protein has hampered understanding the molecular mechanism that these variants utilize to cause diseases. Consequently, this requires comparative investigation of the dynamics of the wild-type and the variant protein at the atomistic detail to understand the impact of the mutation on the structure and dynamics of the protein. In this study, the structural and dynamical properties of one such β -arrestin2 variant, which was shown to associate with high pathogenic score, were investigated using molecular dynamics simulation. Detailed investigation of the trajectories showed that the “short helix” and the “gate loop”, both of which were involved in the activation mechanism of arrestin, were more flexible in the variant and wild-type β -arrestin2 but not in wild-type arrestin1. Moreover, the “finger loop” was also more flexible in the variant such that it was displaced from the N-domain and adopted the “upward position” which is required for receptor binding. Cumulatively, all these local structural rearrangements help the single nucleotide variant adopt active-like domain rotation without perturbing the “polar core”. Interestingly; wild-type β -arrestin2 could sample both active and inactive domain rotation angles. On the other hand, wild-type arrestin1 was more rigid and could not sample active-like domain rotation angle. All these findings were in line with the preference of arrestin subtypes toward receptor phosphorylation. That is is to say, arrestin1 can only bind to activated and phosphorylated rhodopsin, whereas β -arrestin2 can bind to phosphorylated and nonphosphorylated GPCRs depending on the type of the receptor.

From a biological perspective, the capability of A248T mutant for undergoing activation independent of the phosphorylated receptor might lead to either abnormal desensitization of the receptor or overactivation of arrestin-mediated signaling pathways which might be one of the underlying of causes of cancer. Therefore, the A248T mutant can serve as a model system to understand properties of arrestin constructs that can be activated independent of receptor phosphorylation and hence help development of strategies to combat with constitutively active forms of arrestin that might be involved in crucial diseases like cancer.
